# Total Rocuronium Requirement During Intermittent Bolus Versus Continuous Infusion for Deep Neuromuscular Block in Elective Abdominal Cancer Surgery: A Randomized Equivalence Trial

**DOI:** 10.3390/jcm15166135

**Published:** 2026-08-07

**Authors:** Ju Deok Kim, Ji Wook Kim, Hyung Joo Chung, Sie Jeong Ryu, Doo Sik Kim, Seungbo Seo, Donghee Kang

**Affiliations:** Department of Anesthesiology and Pain Medicine, Kosin University Gospel Hospital, 262 Gamcheon-ro, Seo-gu, Busan 49267, Republic of Korea; uamyfriends@kosin.ac.kr (J.D.K.); jwkim@kosin.ac.kr (J.W.K.); dmadjejs@kosin.ac.kr (H.J.C.); siejeong@kosinmed.or.kr (S.J.R.); kdsshj@kosin.ac.kr (D.S.K.); ks250552@kosinmed.or.kr (S.S.)

**Keywords:** rocuronium, neuromuscular blockade, neuromuscular monitoring, intravenous infusions, intravenous injections, sugammadex

## Abstract

**Background/Objectives:** This study compared rocuronium requirements, duration of deep neuromuscular blockade (NMB), and recovery profiles between intermittent bolus and continuous infusion administration. **Methods:** This prospective, single-center, randomized equivalence trial enrolled 72 adults undergoing elective abdominal cancer surgery under sevoflurane–remifentanil anesthesia. The per-protocol analysis included 67 patients (Group B, *n* = 34; Group CI, *n* = 33); five were excluded after neuromuscular monitoring failures precluded primary-outcome calculation. Group B received rocuronium 0.15 mg/kg at post-tetanic count (PTC) ≥ 4. Group CI received 0.3 mg/kg/h after intubation, titrated to maintain PTC ≤ 3. The primary outcome was body weight- and time-normalized total rocuronium requirement, including the intubating dose; the equivalence margin was ±0.13 mg/kg/h. **Results:** Rocuronium requirements were 0.84 ± 0.21 mg/kg/h in Group B and 0.85 ± 0.26 mg/kg/h in Group CI (mean difference, 0.010; 90% CI, −0.087 to 0.108), within the prespecified equivalence margin. A post hoc sensitivity analysis excluding the intubating dose showed no significant difference (0.38 ± 0.14 vs. 0.37 ± 0.13 mg/kg/h; mean difference, −0.012; 95% CI, −0.075 to 0.051; *p* = 0.707). Deep NMB duration (127 ± 43 vs. 132 ± 65 min; *p* = 0.687) and recovery after sugammadex did not differ significantly. Six Group CI patients (18.2%) required rescue boluses. **Conclusions:** The strategies yielded equivalent normalized total rocuronium requirements within the prespecified margin. Recovery outcomes did not differ significantly; however, the infusion protocol required rescue boluses in some patients. PTC-guided individualization is therefore needed.

## 1. Introduction

Rocuronium is a non-depolarizing neuromuscular blocking agent (NMBA) commonly used to facilitate tracheal intubation and provide muscle relaxation during surgery [1]. It can be administered either intermittently as bolus injections or continuously via infusion to maintain neuromuscular blockade (NMB) [2,3]. The method of administration may influence blockade stability, cumulative drug consumption, and recovery characteristics [4,5,6]. While intermittent bolus administration is simple and widely used, it may lead to fluctuations in the depth of NMB due to the variable dosing intervals. In contrast, continuous infusion provides more consistent drug delivery and has been proposed to maintain a more stable pharmacokinetic profile by achieving a balance between drug administration and elimination at steady state [2,3]. However, continuous infusion may require a higher total dose, raising concerns regarding drug accumulation and delayed recovery. Thus, whether these potential pharmacokinetic advantages translate into clinically meaningful benefits over intermittent bolus administration under quantitative neuromuscular monitoring remains unclear.

Numerous studies have investigated optimal rocuronium dosing regimens and monitoring techniques for moderate NMB [6,7,8,9]. However, evidence remains limited regarding the comparative efficacy and dose requirements of intermittent bolus versus continuous infusion, particularly in the context of deep NMB [4,5]. Deep NMB, often defined as a post-tetanic count (PTC) ≤3, has been associated with improved surgical conditions in certain procedures and is increasingly used in abdominal and laparoscopic surgeries. The optimal strategy for maintaining deep NMB may have important clinical implications beyond achieving adequate surgical conditions. Excessive or prolonged exposure to rocuronium may delay recovery and increase the risk of residual NMB, whereas insufficient blockade may compromise the surgical field and require additional rescue dosing. Furthermore, the choice of administration strategy may have implications for reversal strategies, including sugammadex administration, and may affect intraoperative neuromuscular management. Therefore, determining whether continuous infusion provides meaningful advantages over intermittent bolus administration during deep NMB remains clinically relevant. However, the impact of administration strategy on rocuronium requirements and the maintenance of deep NMB under quantitative monitoring has not been fully established.

We hypothesized that the total perioperative rocuronium requirement, including the intubating dose and subsequent maintenance doses required to maintain deep NMB, would be equivalent between intermittent bolus administration and continuous infusion. We therefore conducted a randomized equivalence trial to compare the total perioperative rocuronium requirement between the two administration strategies.

## 2. Materials and Methods

### 2.1. Study Design

This single-center, prospective, randomized equivalence trial was designed to compare the total body weight- and time-normalized rocuronium requirements (mg/kg/h), including the intubating dose, between patients receiving intermittent bolus administration and continuous infusion for maintenance of deep NMB. The equivalence margin was predefined as ±0.13 mg/kg/h, corresponding to approximately 15% of the expected mean requirement. In the clinical context of quantitative neuromuscular monitoring and sugammadex reversal, a difference within this range was considered unlikely to alter the intraoperative titration decisions, reversal strategy, or recovery management. This threshold was determined according to the established principles for equivalence trials, which recommend that the margin should represent the largest difference that would not be considered clinically relevant [10]. The primary analysis was conducted in the per-protocol population. Rescue bolus administration was considered a protocol-defined intervention and not a reason for exclusion. All randomized patients were included in the per-protocol analysis except those in whom the primary outcome could not be calculated because of intraoperative technical failures in quantitative neuromuscular monitoring.

This study was approved by the Institutional Review Board of Kosin University Gospel Hospital (approval no. KUGH-2021-07-48). The study was registered with the Clinical Research Information Service (KCT0006904) before patient recruitment. All study procedures were conducted in accordance with the Declaration of Helsinki of 1975, as revised in 2013, and written informed consent was obtained from all patients before enrollment. This study included patients who underwent surgery at our hospital between December 2021 and April 2022.

### 2.2. Study Population

This study enrolled 72 patients aged 18–65 years with an American Society of Anesthesiologists physical status of I or II who underwent elective gastrointestinal, colorectal, or hepatobiliary cancer surgery under general anesthesia. The exclusion criteria were as follows: body mass index ≥ 30 kg/m^2^, current pregnancy, cerebrovascular disease, renal disease (creatinine clearance < 60 mL/min), liver dysfunction, neuromuscular disease, history of difficult endotracheal intubation, malignant hyperthermia, and drug allergy. Patients who were taking drugs known to interact with rocuronium during surgery (including antibiotics, metronidazole, phenytoin, carbamazepine, diuretics, monoamine oxidase inhibitors, calcium channel blockers, corticosteroids, and magnesium ions) were also excluded.

### 2.3. Randomization and Masking

The allocation sequence was generated by an independent statistician using a computer-generated permuted block randomization sequence with a block size of four and a 1:1 allocation ratio. Group assignments were placed in sequentially numbered, opaque, sealed, tamper-evident envelopes to ensure allocation concealment. The participants were screened and enrolled by a study investigator who had no access to the randomization sequence or envelopes.

Immediately before induction of anesthesia, the attending anesthesiologist, who was not involved in sequence generation or patient enrollment, opened the next envelope and implemented the assigned intervention. Because of the nature of the intervention, the attending anesthesiologist responsible for anesthetic management could not be blinded to group allocation. A second anesthesiologist independently recorded quantitative neuromuscular monitoring measurements and intraoperative outcomes but was not involved in rocuronium administration, infusion-rate adjustment, or rescue bolus decisions. Outcome data were obtained from quantitative neuromuscular monitoring, prospectively recorded perioperative data, and surgeon satisfaction ratings, as applicable.

Surgeons were unaware of treatment allocation and were not informed of intraoperative neuromuscular monitoring data.

### 2.4. Anesthesia Regimen

No preoperative medication was administered to any patient. After entering the operating room, the patient was attached to basic monitors (electrocardiogram, peripheral pulse oximetry, and noninvasive blood pressure measurement). A bispectral index (BIS) Quatro sensor™ (Covidien, Mansfield, MA, USA) was applied to the patient’s forehead to monitor hypnotic depth. An electromyography (EMG)-based neuromuscular monitor (TwitchView™; Blink Device Company, Seattle, WA, USA) was applied to the arm contralateral to the noninvasive blood pressure cuff according to the manufacturer’s instructions, and monitoring was initiated after loss of consciousness [11].

Anesthesia was induced by intravenous injection of propofol (2 mg/kg), remifentanil (1 µg/kg), and rocuronium (0.9 mg/kg). After loss of consciousness, tracheal intubation was performed once a train-of-four (TOF) count of 0 was confirmed on the TwitchView™ monitor. Mechanical ventilation was initiated with a fraction of inspired oxygen of 0.5, tidal volume of 6 mL/kg, respiratory rate of 14 breaths/min, and positive end-expiratory pressure of 6 cmH_2_O. The respiratory rate was adjusted to maintain the end-tidal carbon dioxide pressure between 35 and 40 mmHg. Anesthesia was maintained with continuous remifentanil infusion and 2.0% sevoflurane. To maintain a BIS of 40–60 during surgery, the remifentanil administration rate was adjusted; however, if the BIS could not be maintained within this target range, the sevoflurane concentration was adjusted between 1.8% and 2.2%.

### 2.5. Intervention

After endotracheal intubation, a TOF count of 0 was confirmed, and the TOF mode was switched to the post-tetanic count (PTC) mode. The PTC was measured at 5 min intervals, which is the minimum interval allowed by the TwitchView™ monitor. In this study, we attempted to maintain deep NMB of PTC ≤ 3 during surgery in both groups. Rocuronium was administered for deep NMB after endotracheal intubation in each study group as follows:

Intermittent bolus group (Group B): rocuronium bolus 0.15 mg/kg was administered at PTC ≥ 4.

Continuous infusion group (Group CI): After endotracheal intubation, rocuronium was continuously administered at a rate of 0.3 mg/kg/h. Subsequently, the infusion rate was adjusted within a range of 0.2–0.6 mg/kg/h to maintain PTC ≤ 3. When the measured PTC increased from the previous PTC, the infusion rate was increased by 0.1 mg/kg/h, and when the measured PTC decreased from the previously measured value, the infusion rate was decreased by 0.1 mg/kg/h. The infusion rate was maintained as long as the measured PTC remained unchanged. When the PTC was ≥4, the infusion rate was increased to 0.6 mg/kg/h. However, if PTC ≥ 4 was observed three times in a row despite increasing the infusion rate, a rocuronium bolus of 0.15 mg/kg was administered as rescue treatment.

In both groups, if it was judged after the main procedure that continuation of deep NMB during surgery was unnecessary, the administration of rocuronium was stopped after communicating with the surgeon. Furthermore, to evaluate the recovery pattern from deep NMB, rocuronium administration was stopped in both groups at least 45 min before the end of surgery. To determine the duration of deep NMB, the PTC was measured until the muscle showed a PTC ≥ 4 response. After confirming PTC ≥ 4, the PTC mode was switched back to the TOF mode, and measurements were continuously monitored at 20 s intervals until extubation.

If intraoperative technical failure of the quantitative neuromuscular monitoring device prevented completion of the study protocol or calculation of the primary endpoint, the study intervention was discontinued, and subsequent NMB management was performed according to routine institutional practice. These patients were excluded from the per-protocol analysis.

At the end of the surgery, sevoflurane and remifentanil were discontinued. Sugammadex 4 mg/kg was administered if the TOF count was 0, and 2 mg/kg was administered if the TOF count was ≥1. After confirming a TOF ratio ≥ 0.9, a BIS value ≥ 80, and adequate spontaneous ventilation, the trachea was extubated. Subsequently, patients were transferred to the post-anesthesia care unit (PACU).

### 2.6. Data Collection

#### 2.6.1. Primary Outcome Variable

The primary outcome was body weight- and time-normalized rocuronium requirement for deep NMB, calculated as the total rocuronium dose, including the intubating dose and subsequent maintenance doses, divided by body weight and duration of deep NMB (mg/kg/h).

#### 2.6.2. Secondary Outcome Variables

The duration of deep NMB was defined as the interval from the first confirmation of a PTC of 0 after endotracheal intubation to recovery to PTC ≥ 4 after discontinuation of rocuronium.

The total dose of rocuronium administered during surgery was recorded. This was calculated as the sum of the rocuronium dose for endotracheal intubation and the dose corresponding to the administration method used in each group. To measure the rocuronium dose administered after endotracheal intubation in Group B, the doses and frequencies of bolus administrations when PTC ≥ 4 were recorded. In Group CI, the continuously infused rocuronium dose after endotracheal intubation and the rescue dose administered after three consecutive PTC ≥ 4 measurements were recorded.

After cessation of rocuronium administration for deep NMB, the time required from PTC ≤ 3 to a TOF count of 1 and to extubation was recorded. The TOF count at the time of sugammadex administration, as well as the time required from sugammadex administration to a TOF ratio of 0.9 and to extubation, were also recorded. After surgery, the surgeon’s overall satisfaction with muscle relaxation and the surgical field was rated on a 100-point scale (0, worst; 100, best). Hypoxemia, defined as SpO_2_ < 92%, was recorded from tracheal extubation until 30 min after PACU admission. Pain numeric rating scale (NRS) scores, fentanyl consumption, and the occurrence of nausea, vomiting, and headache in the PACU were also recorded.

#### 2.6.3. Sample Size Calculation

Based on our preliminary pilot study conducted at this institution (unpublished data), the body weight- and time-normalized total rocuronium requirement for deep NMB, including the intubating dose and subsequent maintenance doses, was estimated to be approximately 0.86 ± 0.18 mg/kg/h in patients receiving intermittent bolus administration. The equivalence margin was prespecified as ±0.13 mg/kg/h. This margin was selected based on clinical considerations regarding the potential impact of differences in rocuronium requirements during quantitative neuromuscular monitoring. A difference within this range was considered unlikely to result in clinically meaningful changes in rocuronium exposure or to alter the intraoperative dose-titration decisions, reversal strategy, or recovery management when quantitative neuromuscular monitoring and sugammadex reversal were available. This threshold corresponded to approximately 15% of the expected mean rocuronium requirement and was determined according to the established principles for equivalence trials, which recommend that the margin should represent the largest difference that is considered clinically unimportant [10]. Consistent with this principle, the ICH E9 Statistical Principles for Clinical Trials and ICH E10 Choice of Control Group and Related Issues in Clinical Trials guidelines emphasize that equivalence margins should be based on clinical rather than purely statistical considerations. Thirty-three patients were needed in each group to achieve a statistical power of 80% with a type I error of 0.05 and a type II error of 0.2 [10]. Considering a dropout rate of 10%, 72 patients were included in this study.

#### 2.6.4. Data Analysis and Statistical Methods

All statistical analyses were performed using SPSS version 20.0 software (IBM Corp., Armonk, NY, USA). Equivalence of the primary outcome, the body weight- and time-normalized rocuronium requirement for deep NMB, was assessed using the two one-sided test procedure. Equivalence was concluded if the 90% confidence interval (CI) for the between-group difference, calculated as Group CI minus Group B, was entirely contained within the prespecified equivalence margin of −0.13 to +0.13 mg/kg/h. Additional post hoc sensitivity analyses were performed. First, the six patients in Group CI who required rescue bolus administration were excluded, and the primary outcome was reanalyzed using the same equivalence framework with a 90% CI and the prespecified equivalence margin. Second, the intubating rocuronium dose was excluded, and the maintenance rocuronium requirement after endotracheal intubation, normalized to body weight and the duration of deep neuromuscular blockade (mg/kg/h), was analyzed.

The secondary outcomes were evaluated using conventional statistical tests for between-group comparisons. The normality of continuous variables was evaluated using the Shapiro–Wilk test. The two-tailed unpaired Student’s *t*-test was used to compare normally distributed continuous variables between groups (duration of deep NMB, total rocuronium dose used during surgery, time required from PTC ≤ 3 to a TOF count of 1 and to extubation, time required from sugammadex administration to a TOF ratio of 0.9 and to extubation, surgeon satisfaction score, and NRS score in the PACU), and the data were expressed as the mean and standard deviation (SD).

Categorical data, such as the TOF count at the time of sugammadex administration and adverse events in the PACU, were examined using the chi-square test or Fisher’s exact test, as appropriate. Statistical significance was set at *p* < 0.05.

## 3. Results

**Figure 1:**
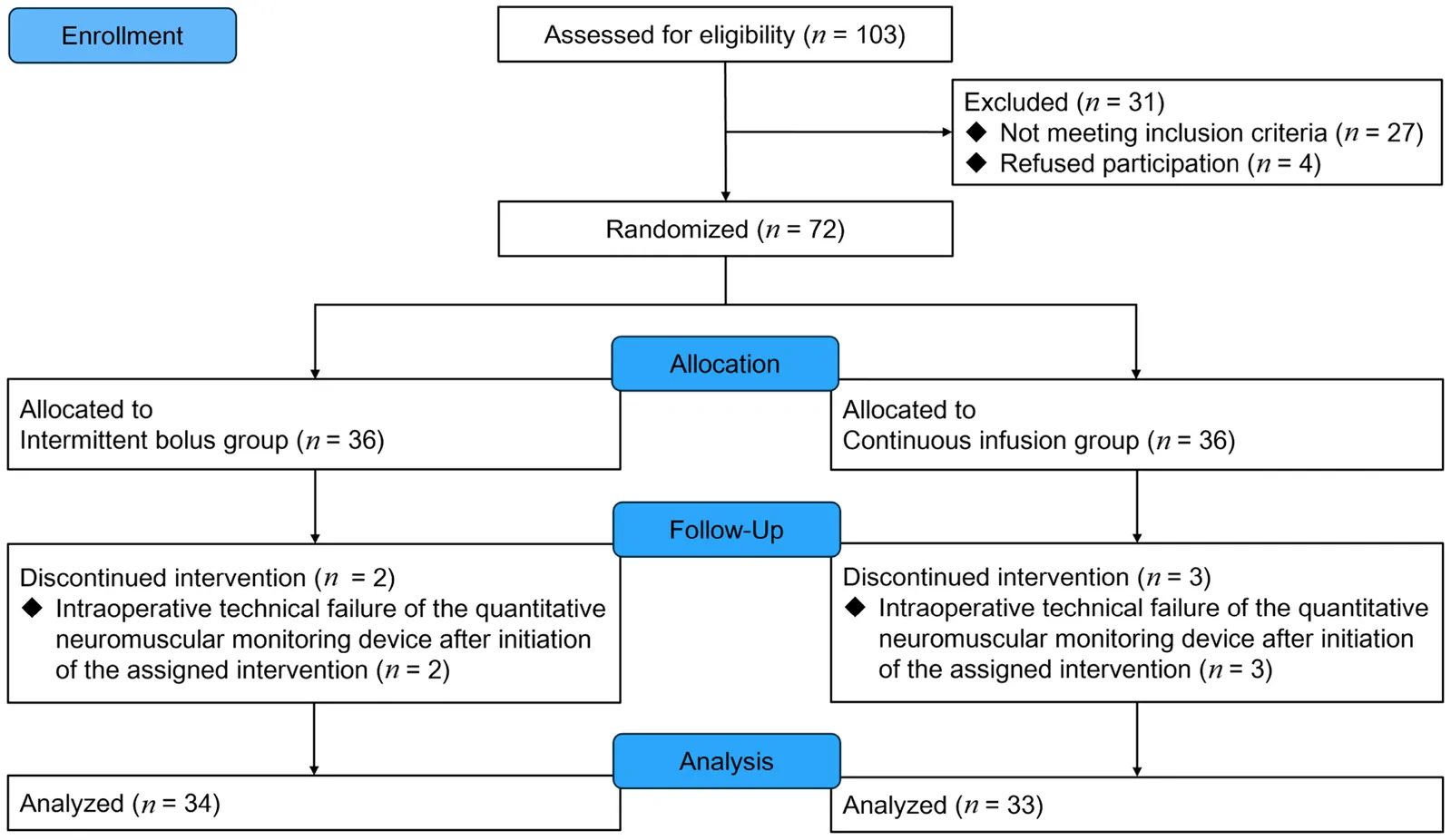
Flowchart of study participants.

Of the 72 randomized patients, five were excluded from the per-protocol analysis because of technical failures in neuromuscular monitoring that precluded the calculation of the primary outcome. The per-protocol analysis population included 67 patients (34 in the intermittent bolus group and 33 in the continuous infusion group). Technical failures of the neuromuscular monitor occurred either before study measurements were obtained or during intraoperative monitoring, precluding calculation of the primary outcome (Figure 1). Baseline patient characteristics, including age, sex, height, and weight, were comparable between the two groups. There were no significant between-group differences in the durations of anesthesia or surgery, intraoperative fluid volume, remifentanil consumption, or urine output (Table 1).

**Figure 2:**
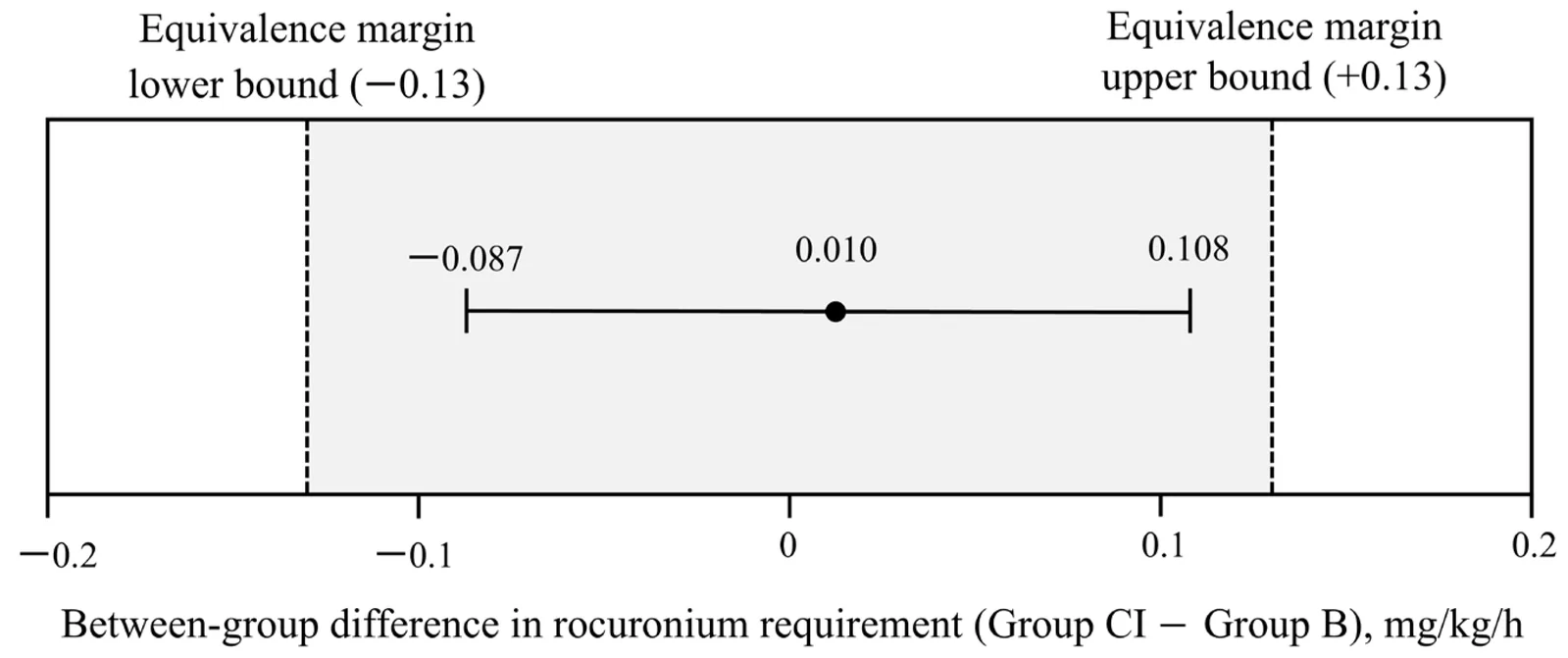
Mean between-group difference in body weight- and time-normalized total rocuronium requirement for deep neuromuscular blockade, calculated as Group CI minus Group B. The filled circle represents the point estimate, and the horizontal error bar represents the 90% confidence interval. The shaded area indicates the prespecified equivalence range of −0.13 to +0.13 mg/kg/h.

The primary outcome, the body weight- and time-normalized rocuronium requirement for deep NMB, was 0.84 ± 0.21 mg/kg/h in Group B and 0.85 ± 0.26 mg/kg/h in Group CI. The mean between-group difference, calculated as Group CI minus Group B, was 0.010 mg/kg/h, with a 90% CI of −0.087 to 0.108 mg/kg/h. The 90% CI was entirely contained within the prespecified equivalence margin of −0.13 to +0.13 mg/kg/h; therefore, equivalence between continuous infusion and intermittent bolus administration was demonstrated (Figure 2). In a post hoc sensitivity analysis excluding the six patients who required rescue bolus administration, the normalized rocuronium requirement in the remaining Group CI patients was 0.79 ± 0.21 mg/kg/h. The mean difference (Group CI minus Group B) was −0.047 mg/kg/h (90% CI, −0.137 to 0.044 mg/kg/h). Because the 90% CI was not entirely contained within the prespecified equivalence margin, equivalence was not demonstrated in this restricted post hoc analysis.

Neither the duration of deep NMB nor the total rocuronium dose differed significantly between groups (Table 2).

As an additional post hoc sensitivity analysis, the maintenance rocuronium requirement excluding the intubating dose was compared between groups. There was no statistically significant difference between the intermittent bolus and continuous infusion groups (0.38 ± 0.14 vs 0.37 ± 0.13 mg/kg/h; mean difference −0.012 mg/kg/h, 95% CI −0.075 to 0.051; *p* = 0.707).

Six patients (18.2%) in Group CI required a total of eight rescue bolus administrations because PTC remained ≥4 on three consecutive measurements despite adjusting the infusion rate according to the predefined protocol. Two patients required rescue bolus administration twice. The individual characteristics of the six patients who required rescue bolus administration are presented in Supplementary Table S1. Descriptive comparison with the remaining patients in Group CI revealed no clear distinguishing pattern in the clinical and perioperative characteristics evaluated.

Table 3 shows that no statistically significant between-group differences were observed in the recovery variables or time to extubation after discontinuation of rocuronium. At the end of the surgery, three patients in Group B and five patients in Group CI exhibited a TOF count of 0, and all other patients showed a TOF count ≥ 1.

No prespecified PACU adverse events were observed in either group. No statistically significant between-group difference was detected in the surgeon satisfaction score (Table 4).

## 4. Discussion

In this randomized equivalence trial, the body weight- and time-normalized total rocuronium requirement, including the intubating dose, was equivalent between intermittent bolus administration and continuous infusion within the prespecified margin in patients undergoing elective gastrointestinal, colorectal, or hepatobiliary cancer surgery. No statistically significant between-group differences were observed in the duration of deep NMB or neuromuscular recovery outcomes after sugammadex reversal. However, 18.2% of the patients in the continuous infusion group required rescue bolus administration despite protocol-based infusion-rate adjustment, indicating that the predefined infusion protocol did not maintain the target depth of NMB in all patients. This finding may reflect the infusion-rate adjustment algorithm, monitoring interval, or interindividual variability in rocuronium requirements, rather than an inherent limitation of continuous infusion itself. In the post hoc sensitivity analysis excluding the six patients who required rescue bolus administration, equivalence was not demonstrated as the 90% CI was not entirely contained within the prespecified equivalence margin. However, because rescue bolus administration was prespecified as part of the continuous-infusion protocol, excluding patients based on this post-randomization treatment event limits the interpretability of the analysis. Accordingly, the findings should be regarded as exploratory and interpreted alongside the primary equivalence analysis.

Because both groups received the same intubating dose, inclusion of this dose in the primary outcome could theoretically attenuate differences in maintenance rocuronium requirements between the groups. Therefore, we performed an additional post hoc sensitivity analysis excluding the intubating dose. No statistically significant difference in maintenance rocuronium requirement was observed between the two groups, and the findings were generally consistent with those of the primary analysis. However, because the prespecified equivalence margin was defined only for the primary outcome, the maintenance-only analysis should be interpreted as supportive rather than confirmatory. The equivalence margin was determined before study initiation based on pilot data and clinical consideration because no validated equivalence margin for total rocuronium requirement has been established. The prespecified margin of ±0.13 mg/kg/h represented approximately 15% of the expected mean rocuronium requirement. When translated post hoc into an absolute dose using the mean body weight and duration of deep NMB observed in this cohort, this margin corresponded to approximately 18.1 mg of rocuronium per case, or approximately 1.9 protocol-specified maintenance boluses of 0.15 mg/kg. Although greater rocuronium exposure may prolong recovery from neuromuscular blockade, the actual between-group difference in rocuronium requirement was minimal and was accompanied by similar recovery outcomes after sugammadex reversal. This post hoc conversion provides additional clinical context for interpreting the magnitude of the prespecified equivalence margin, although further studies may help refine clinically relevant equivalence thresholds for rocuronium requirement.

Several comparative studies have examined the administration methods of neuromuscular blocking agents, mainly in the context of moderate NMB. However, evidence remains limited regarding the rocuronium dose required for deep NMB [4,5,12]. In the present study, the body weight- and time-normalized total rocuronium requirement, including the intubating dose, was 0.84 mg/kg/h in Group B and 0.85 mg/kg/h in Group CI, supporting equivalence between the two strategies. This finding differs from those of previous studies on moderate NMB, in which continuous infusion required a lower dose than intermittent bolus administration. Choi et al. reported that the rocuronium dose for continuous infusion was significantly lower than that for intermittent bolus administration (4.9 µg/kg/min vs. 6.1 µg/kg/min, respectively) to maintain moderate NMB below a TOF count of 2 in children [4]. These findings suggest that continuous infusion can produce a similar moderate NMB effect with a lower rocuronium dose than intermittent bolus administration. In a study using cisatracurium in adults, another NMBA, continuous infusion required a lower dose than intermittent bolus administration to maintain a twitch response of less than 20% following neuromuscular stimulation [5]. Furthermore, these studies suggested that continuous infusion can maintain the desired degree of NMB more stably than intermittent bolus administration.

The major difference between the present and previous studies is the depth of targeted NMB during surgery. Neuromuscular transmission has a margin of safety [13]. For moderate NMB, approximately 70–80% of acetylcholine receptors in the postsynaptic membrane must be occupied by the NMBA [14]. Therefore, the dose-sparing effect of continuous infusion reported in previous moderate NMB studies may have reflected the amount of rocuronium required to maintain this relatively lower level of receptor occupancy after the initial intubating dose. In contrast, deep NMB requires approximately 90–95% receptor occupancy, at which point TOF responses can no longer be elicited [15]. Complete neuromuscular block with no PTC response may require at least 95% receptor occupancy. Previous studies on continuous infusion of rocuronium reported infusion rates of 0.65 mg/kg/h for maintaining PTC 0–5 and 0.833 mg/kg/h for maintaining PTC 1–2 [6]. As summarized by Couto et al., the included studies differed in several methodological aspects, including patient characteristics, duration and type of surgery, anesthetic regimens, and rocuronium infusion protocols. In the post hoc sensitivity analysis excluding the intubating dose, the normalized post-intubation maintenance rocuronium requirements were 0.38 mg/kg/h in Group B and 0.37 mg/kg/h in Group CI, compared with normalized total requirements of 0.84 and 0.85 mg/kg/h, respectively, in the primary analysis. This difference reflects the contribution of the 0.9 mg/kg intubating dose to the primary outcome. The remaining differences may also reflect variations in anesthetic management, intubating doses, infusion initiation and dose-adjustment strategies, and target depths of NMB. As noted by Couto et al., rocuronium infusion requirements should be interpreted in the context of the specific clinical setting and dosing protocol used. Thus, the present study provides clinically relevant dose information for maintaining deep NMB during abdominal surgery of approximately 2–3 h under quantitative neuromuscular monitoring.

Rocuronium is a steroidal non-depolarizing neuromuscular blocker with rapid onset and intermediate duration of action [16]. The target of maintaining deep NMB also influenced the dosing protocol used in the present study. In the current study, 0.9 mg/kg of rocuronium was used for endotracheal intubation instead of 0.6 mg/kg, the dose frequently used in previous studies. This dose was selected because 0.6 mg/kg of rocuronium often failed to achieve the intended deep NMB indicated by PTC ≤ 3 after endotracheal intubation. Consequently, an additional dose was often required for deep NMB. Therefore, 0.9 mg/kg was administered during anesthetic induction to achieve deep NMB from the outset. In the continuous infusion group, continuous infusion was initiated immediately after endotracheal intubation without waiting for recovery to PTC ≥ 4. When rocuronium infusion is initiated only after reaching PTC ≥ 4, it may take longer to administer the required rocuronium dose to achieve and maintain the intended deep NMB in the continuous infusion group than in the intermittent bolus group. Therefore, continuous infusion was initiated immediately after endotracheal intubation to avoid a delay in achieving and maintaining the target depth of NMB in Group CI.

This deeper target may also explain why infusion-rate adjustment alone was not sufficient in some patients in the continuous infusion group. In Group CI, a single bolus dose of 0.15 mg/kg was administered when PTC ≥ 4 was observed three times in a row despite adjustment of the infusion rate. The TwitchView™ monitor used in this study can measure PTC only every 5 min. This restriction represents a limitation of currently available monitoring methods for deep NMB, as continuous assessment at intervals shorter than 5 min can lead to inaccurate neuromuscular results. Thus, three consecutive occurrences of PTC ≥ 4 using the TwitchView™ monitor indicate that the targeted deep NMB was not reached for at least 10 min. Therefore, rescue bolus administration was incorporated into the protocol to restore the target depth of NMB when infusion-rate adjustment alone was insufficient. Miller et al. reported that rocuronium is eliminated primarily by the liver and marginally by the kidneys during continuous infusion. Therefore, rocuronium may accumulate, making control within the desired clinical range difficult [17]. Indeed, 6 of the 33 patients in Group CI did not achieve the target deep NMB within 10 min by infusion rate adjustment alone. However, all six patients achieved PTC ≤ 3 after rescue bolus administration. This reflected the predefined rescue protocol, which prioritized infusion rate adjustment before rescue bolus administration rather than immediate correction after a single PTC excursion. The need for rescue bolus administration should be interpreted in the context of the predefined infusion protocol used in this study, including the predefined infusion-rate adjustment range (0.2–0.6 mg/kg/h) and the inherent temporal limitations of PTC monitoring. Accordingly, the need for rescue bolus administration should not necessarily be attributed solely to the pharmacologic characteristics of continuous infusion. Whether alternative infusion rates or adjustment algorithms could reduce the need for rescue boluses warrants further investigation.

The depth of NMB suitable for most general surgical operations is known as moderate NMB with a TOF count of 1–2 [18]. However, deep NMB is sometimes required for surgical convenience [19]. One study reported that maintaining PTC ≤ 5 may prevent hiccups, bucking, and coughing, which have been shown to occur during surgical procedures despite the total abolition of TOF responses [20]. Although it has not yet been established whether the intraoperative beneficial effects of deep NMB lead to better perioperative outcomes, the application of deep NMB improves the surgical environment by sufficiently relaxing the diaphragm muscle and preventing sudden and unexpected movements of the patient during laparoscopic surgery compared to the application of moderate NMB [21,22,23]. The results of our study may help anesthesiologists better understand the total rocuronium requirements, including the intubating dose and maintenance doses, when using intermittent bolus administration or continuous infusion to maintain deep NMB during elective gastrointestinal, colorectal, or hepatobiliary cancer surgery.

Quantitative neuromuscular monitoring is essential for evaluating deep NMB during general anesthesia [24,25]. In the present study, EMG-based monitoring (TwitchView™) was used instead of acceleromyography (AMG). AMG requires unrestricted thumb movement, and accurate measurements may therefore be difficult when the arm is tucked during laparoscopic or robotic surgery [25]. In contrast, EMG can assess neuromuscular function without requiring thumb movement [26]. A TOF ratio ≥ 0.9 should be confirmed before tracheal extubation after NMBA administration, including after deep NMB. Although neuromuscular monitoring failed in five enrolled patients, possibly because of equipment malfunction or poor electrode contact, EMG-based monitoring enabled assessment of NMB depth across various surgical and anesthetic settings, including procedures performed with the arm tucked. Objective monitoring is particularly important during intermittent bolus administration because redosing should be guided by PTC responses rather than performed empirically at fixed intervals.

The application of deep NMB during surgery may be burdensome to anesthesiologists because of concerns regarding prolonged recovery and residual NMB [27]. Although recovery after sugammadex administration was numerically longer in Group CI, no statistically significant between-group differences were observed in the time from PTC ≤ 3 to a TOF count ≥ 1 or from sugammadex administration to extubation. These findings are consistent with previous studies showing that recovery to TOF ratios of 0.7 and 0.9 did not differ between administration methods [4].

In the present study, the time from sugammadex administration to recovery to a TOF ratio of 0.9 was 221 ± 85 s in Group B and 258 ± 80 s in Group CI. The numerically longer recovery in Group CI may partly reflect differences in the timing of the final rocuronium administration. In Group CI, infusion was generally continued until the protocol-defined discontinuation point, whereas the final bolus in Group B may have been administered earlier depending on the PTC values. Consequently, the residual effect of rocuronium at the time of reversal may have been greater in Group CI despite similar overall rocuronium exposure. The numerically higher surgeon satisfaction in Group CI may likewise be consistent with more sustained muscle relaxation; however, this interpretation remains speculative because the timing of the final rocuronium administration and the proportion of time within the target PTC range were not analyzed.

These recovery times were slightly longer than the 2.7 min reported in a previous study, in which sugammadex 2.0 mg/kg was administered at a TOF count of 2 [28]. According to the study protocol, rocuronium administration was stopped at least 45 min before the end of surgery. Because most patients had a TOF count ≥ 1 at the time of sugammadex administration, the depth of NMB at reversal may have differed from that in previous studies, making direct comparison of recovery times difficult [29]. Overall, residual NMB-related complications were not observed in either group.

### Study Limitations

This study has several limitations. First, PTC measurements were limited by the technical characteristics of the neuromuscular monitoring devices. The TwitchView™ monitor allows PTC assessment at a minimum interval of 5 min; therefore, more frequent measurements were not performed because they may have resulted in unreliable neuromuscular assessments. Consequently, rapid changes in the depth of NMB may not have been detected immediately or promptly reflected in rocuronium dose adjustments. This temporal limitation may have affected titration, particularly in the continuous infusion group, in which infusion-rate adjustments were made according to intermittently measured PTC values. Second, this study was a single-center study conducted in a relatively homogeneous population of patients aged 18–65 years with an American Society of Anesthesiologists physical status I or II who underwent elective gastrointestinal, colorectal, or hepatobiliary cancer surgery. Therefore, the findings cannot be generalized to elderly patients, patients with obesity, patients with renal or hepatic impairment, those undergoing emergency surgery, or other high-risk surgical populations, including those undergoing thoracic or neurosurgical procedures, where the pharmacokinetic and pharmacodynamic profiles of rocuronium may differ substantially. Third, the potential influence of inhalational anesthetic exposure on NMB cannot be completely excluded, as anesthesia was maintained with sevoflurane rather than with total intravenous anesthesia [30]. Although sevoflurane concentration was standardized according to the study protocol and maintained within a predefined range (1.8–2.2%) in both groups, individual end-tidal sevoflurane concentrations were not recorded for the analysis. Therefore, subtle differences in volatile anesthetic exposure between the groups cannot be completely excluded. Finally, detailed assessments of deep NMB maintenance characteristics, including time within the target PTC range and frequency of PTC excursions, were not prespecified outcomes in this study and were therefore not evaluated. Further studies specifically designed to evaluate these parameters are warranted.

Among adult patients undergoing elective abdominal cancer surgery under sevoflurane anesthesia, intermittent bolus administration and continuous infusion resulted in equivalent body weight- and time-normalized total rocuronium requirements, including the intubating dose, within the prespecified equivalence margin. No statistically significant differences were observed between groups in the secondary outcomes. Both strategies were feasible for intraoperative deep NMB management under quantitative EMG-based monitoring within the protocols evaluated in this study. However, rescue bolus administration was required in a subset of patients receiving continuous infusion. These findings suggest that, when a similar PTC-guided continuous infusion protocol is used, rocuronium dosing may need to be individualized according to PTC responses, with supplemental boluses administered when necessary.

## Figures and Tables

**Table 1:** Baseline characteristics and surgical data of participants.

	Group B (*n* = 34)	Group CI (*n* = 33)	*p*
Sex (M/F)	22/12	19/14	0.782
Age (years)	56.1 ± 8.1	57.6 ± 6.0	0.397
Height (cm)	165.6 ± 7.8	165.2 ± 8.5	0.849
Weight (kg)	64.8 ± 10.2	64.5 ± 8.7	0.878
Body mass index (kg/m^2^)	23.7 ± 2.7	23.7 ± 2.1	0.903
Duration of surgery (min)	143.0 ± 44.7	140.3 ± 65.6	0.841
Duration of anesthesia (min)	179.1 ± 50.0	174.2 ± 66.0	0.725
Fluid infusion (mL)	1127 ± 365	1056 ± 284	0.381
Urine output (mL)	264 ± 156	274 ± 146	0.763
Remifentanil infusion (µg)	951 ± 328	919 ± 468	0.682
Type of surgery			0.937
Gastrointestinal surgery	20	18	
Colorectal surgery	12	13	
Hepatobiliary surgery	2	2	
Surgical approach			0.728
Laparoscopic	25	23	
Open	9	10	

Group B: rocuronium bolus group (0.15 mg/kg); Group CI: rocuronium continuous infusion group (0.2–0.6 mg/kg/h).

**Table 2:** Rocuronium requirements, consumption, and duration of deep neuromuscular blockade.

	Group B (*n* = 34)	Group CI (*n* = 33)	Mean Difference (CI)	*p* Value
Total rocuronium requirement for deep NMB (mg/kg/h)	0.84 ± 0.21	0.85 ± 0.26	0.010 (−0.087 to 0.108)	N/A
Sensitivity analysis excluding rescue bolus patients ^†^ (mg/kg/h)	0.84 ± 0.21	0.79 ± 0.21 (*n* = 27)	−0.047 (−0.137 to 0.044)	N/A
Maintenance rocuronium requirement for deep NMB ^‡^ (mg/kg/h)	0.38 ± 0.14	0.37 ± 0.13	−0.012 (−0.075 to 0.051)	0.707
Total rocuronium consumption (mg)	108.9 ± 31.9	108.1 ± 31.8	−0.8 (−16.1 to 14.6)	0.924
Rocuronium dose for intubation (mg)	57.7 ± 9.2	57.7 ± 7.6	0.0 (−4.1 to 4.1)	0.984
Rocuronium dose after intubation (mg)	51.1 ± 26.4	50.4 ± 29.0	−0.7 (−14.0 to 12.6)	0.918
Total bolus dose (mg)	51.1 ± 26.4	N/A	−	−
Continuous infusion dose (mg)	N/A	48.1 ± 29.5	−	−
Patients requiring rescue bolus administration, *n* (%)	N/A	6 (18.2)	−	−
Duration of deep NMB (min)	127 ± 43	132 ± 65	5.0 (−20.9 to 31.5)	0.687

Data are expressed as mean ± SD or number (%). Mean differences were calculated as Group CI minus Group B. For the primary equivalence outcome and the post hoc sensitivity analysis excluding rescue bolus patients, mean differences are presented with 90% CIs; for other continuous outcomes, 95% CIs are presented. ^†^ Post hoc sensitivity analysis excluding the six patients in Group CI who required rescue bolus administration. ^‡^ Sensitivity analysis excluding the intubating dose. Group B, intermittent bolus administration group; Group CI, continuous infusion group; NMB, neuromuscular blockade; CI, confidence interval; N/A, not applicable; SD, standard deviation.

**Table 3:** Data related to neuromuscular blockade recovery.

	Group B (*n* = 34)	Group CI (*n* = 33)	95% CI	*p*
Patient’s muscle relaxation at the end of surgery (TOF count 0/TOF count ≥ 1)	3/31	5/28		0.476
Time required from PTC ≤ 3 to a TOF count of 1 (min)	22.4 ± 7.6	23.3 ± 8.8	−3.6–5.8	0.649
Time required from PTC ≤ 3 to extubation (min)	42.2 ± 15.3	36.4 ± 14.2	−13.1–1.38	0.111
Time required from sugammadex to TOF 0.9 (s)	221 ± 85	258 ± 80	−4.0–78.4	0.076
Time required from sugammadex to extubation (s)	324 ± 95	342 ± 92	−28.9–65.5	0.441

Data are expressed as mean ± SD or number. The 95% CIs correspond to mean differences calculated as Group CI minus Group B. Group B, intermittent bolus administration group (0.15 mg/kg); Group CI, continuous infusion group (0.2–0.6 mg/kg/h); CI, confidence interval; PTC, post-tetanic count; SD, standard deviation; TOF, train-of-four.

**Table 4:** Patients’ conditions in the post-anesthesia care unit, adverse events, and surgeon satisfaction score.

	Group B (*n* = 34)	Group CI (*n* = 33)	95% CI	*p*
NRS score in the PACU	6.1 ± 1.8	6.3 ± 1.3	−0.60–0.93	0.659
Fentanyl administered in the PACU (µg)	101.4 ± 28.0	104.8 ± 26.9	−10.0–16.9	0.611
Hypoxemia in PACU, *n* (%)	0 (0)	0 (0)	—	—
Nausea, *n* (%)	0 (0)	0 (0)	—	—
Vomiting, *n* (%)	0 (0)	0 (0)	—	—
Headache, *n* (%)	0 (0)	0 (0)	—	—
Surgeon satisfaction score	94.3 ± 5.9	96.7 ± 5.3	−0.33–5.12	0.084

Data are expressed as mean ± SD or number (%). The 95% CIs correspond to mean differences calculated as Group CI minus Group B. Hypoxemia was defined as oxygen saturation < 92% from extubation to 30 min in the PACU. Surgeon satisfaction was scored on a scale from 0 (worst) to 100 (best). Group B, intermittent bolus administration group; Group CI, continuous infusion group; CI, confidence interval; NRS, numeric rating scale; PACU, post-anesthesia care unit; SD, standard deviation.

## Data Availability

The datasets generated during the current study are available from the corresponding author on reasonable request.

## Supplementary Materials

The following supporting information can be downloaded at https://www.mdpi.com/article/10.3390/jcm15166135/s1, Table S1: Characteristics of patients in the continuous infusion group who required rescue bolus administration.

## Abbreviations

The following abbreviations are used in this manuscript:

BIS	Bispectral index
CI	Confidence interval
NMB	Neuromuscular blockade
NRS	Numeric rating scale
PACU	Post-anesthesia care unit
PTC	Post-tetanic count
TOF	Train-of-four
