# Supplementary material for: Total Rocuronium Requirement During Intermittent Bolus Versus Continuous Infusion for Deep Neuromuscular Block in Elective Abdominal Cancer Surgery: A Randomized Equivalence Trial
Source: J Clin Med. 2026 Aug 7;15(16):6135. doi: 10.3390/jcm15166135 (PMC13513413; doi:10.3390/jcm15166135)
Supplement: Supplementary file 1 [file jcm-15-06135-s001.zip › jcm-4408746-supplementary.pdf]

Supplementary Table S1. Characteristics of patients in the continuous infusion group who required rescue bolus administration

Characteristic	Patient 1	Patient 2	Patient 3	Patient 4	Patient 5	Patient 6
Age (yr)	63	52	65	58	44	62
Sex	Male	Female	Male	Female	Female	Male
BMI (kg/m²)	27.08	20.63	24.50	20.49	24.26	23.98
ASA physical status	2	2	2	2	2	2
Surgical procedure	TG + LND	TG + LND	LAR	LAR	TLDG	LAR
Duration of deep NMB (min)	143	97	72	105	95	135
Total rocuronium requirement for deep NMB (mg/kg/h)	0.631	1.065	1.767	1.011	1.065	1.020
Maintenance rocuronium requirement† (mg/kg/h)	0.248	0.498	0.628	0.489	0.501	0.620
Number of rescue bolus administrations	1	2	1	1	2	1

Data are presented for individual patients in the continuous infusion group who required rescue bolus administration. † Post hoc sensitivity analysis excluding the intubating dose. BMI, body mass index; ASA, American Society of Anesthesiologists; NMB, neuromuscular blockade; TG, total gastrectomy; LND, lymph node dissection; LAR, low anterior resection; TLDG, totally laparoscopic distal gastrectomy. Formal statistical comparisons were not performed because of the small number of patients requiring rescue bolus administration.
